# Comparison of 1D and 3D volume measurement techniques in NF2-associated vestibular schwannoma monitoring

**DOI:** 10.1038/s41598-025-85386-4

**Published:** 2025-01-17

**Authors:** Isabel Gugel, Nuran Aboutaha, Bianca Pfluegler, Ulrike Ernemann, Martin Ulrich Schuhmann, Marcos Tatagiba, Florian Grimm

**Affiliations:** 1 Department of Neurosurgery, Centre of Neurofibromatosis and Schwannomatosis, Centre for Rare Diseases, University Hospital Tübingen, Tübingen, Germany; 2 Department of Neurosurgery, University Hospital Tübingen, Tübingen, Germany; 3 Department of Neuroradiology, University Hospital Tübingen, Tübingen, Germany; 4 Department of Neurosurgery, Centre of Neurofibromatosis and Schwannomatosis, Centre for Rare Diseases, Division of Pediatric Neurosurgery, University Hospital Tübingen, Tübingen, Germany

**Keywords:** Vestibular Schwannoma, Neurofibromatosis type 2 associated Schwannomatosis, Segmented volumetric analysis, Tumor Growth, Linear measurements, Cancer genetics, Cancer in the nervous system, Outcomes research, Data acquisition, Image processing, Software

## Abstract

To compare 1D (linear) tumor volume calculations and classification systems with 3D-segmented volumetric analysis (SVA), focusing specifically on their effectiveness in the evaluation and management of NF2-associated vestibular schwannomas (VS). VS were clinically followed every 6 months with cranial, thin-sliced (< 3 mm) MRI. We retrospectively reviewed and used T1-weighted post-contrast enhanced (gadolinium) images for both SVA and linear measurements. 3D-SVA was performed manually or combined with semiautomated segmentation by using axial planes. The maximum linear dimensions (MLD) were determined in three dimensions (anteroposterior, transverse, and craniocaudal planes) using axial and coronal planes. The MLD was cubed (MLD^3^), and orthogonal analysis (OA) was derived to establish comparability with the SVA. The Hannover and Koos classification was used to depict the size ratio in each MRI and tumor. A linear regression model was performed to compare 1D/classification systems to SVA, and the percentage deviation change of MLD^3^ and OA to SVA was established using a one-way multivariate variance analysis. 2586 SVA and 10344 linear measurements were performed in a cohort of 149 NF2 patients and 292 associated VS. All measurement techniques (MLD^3^, OA, KOOS, and Hannover) significantly (and strongly, r^2^ > 0.5) correlated with SVA (*p < 0.001*). The OA showed an even stronger positive correlation than the MLD^3^ to SVA. Smaller classified tumors (T1/T2, K1/K2) exhibited a low-moderate positive correlation (r^2^ = 0.23–0.44) compared to medium-sized (T3, K2/3) and large tumors (T4, K4; r^2^ = 0.54–0.76). Pre- and postoperative MLD^3^ and OA statistically significantly predict SVA (*p* < 0.001), but the postoperative correlation was weaker, particularly for MLD^3^ to SVA values. All analyses showed a large scatter range. In the percentage deviation analysis of MLD^3^ and OA from SVA, small tumors (K1/K2, T1/T2) were overestimated. Compared to the SVA, the MLD^3^ and especially the OA are a time-saving alternative for monitoring the tumor volume of NF2-associated VS. However, the scatter range in small/surgically reduced tumors is enormous. For this reason, they are not recommended for monitoring off-label therapy with Bevacizumab or for treatment decisions depending on a precise assessment of tumor volume and growth. Developing deep learning-based volume determinations in the future is essential to reduce SVA’s time intensity.

**Supplementary Information:**

The online version contains supplementary material available at 10.1038/s41598-025-85386-4.

## Introduction

The development of bilateral vestibular schwannomas (VS) is the hallmark of the tumor predisposition syndrome Neurofibromatosis Type 2 (NF2) related schwannomatosis. VS-related symptoms such as hearing loss, tinnitus, and balance disorders may occur early in the course of the disease and are the major presenting symptoms in adults concerning NF2^1^. Due to VS, which is highly penetrating during the disease, there is a very high risk of bilateral deafness during disease progress. The early evaluation of tumor volume and growth behavior in NF2-associated vestibular schwannoma (VS) by regular, serial cranial magnetic resonance imaging (MRI) is indispensable. Contrast-enhanced CT can be an alternative for patients who cannot undergo MRI. This is generally less susceptible to artifacts caused by hearing implants, for example.

Nevertheless, in NF2 patients, MRI remains the gold standard mainly due to the low risk of radiation exposure. T1-weighted sequences with contrast agents in axial and coronal planes facilitate an optimal evaluation of NF-associated VS. In addition, T2-weighted and Fluid-Attenuated Inversion Recovery (FLAIR) sequences prove valuable in assessing cystic components present within or adjacent to the tumor^2^. Other functional MRI techniques, such as dynamic contrast-enhanced MRI, diffusion tensor imaging (DTI), and spiral ^23^Na-MRI acquisition for total sodium concentration quantification, may also help to evaluate treatment responses following radiosurgery^3,4^ or antiangiogenic therapy^5^.

Imaging is intended to find the right time point of therapy in combination with hearing diagnostics. Depending on tumor size, growth behavior, and hearing function, treatment is predominantly limited to a “wait and scan” strategy, surgery, and off-label use of the vascular endothelial growth factor (VEGF) inhibitor bevacizumab.

Cranial MRI should be performed every 4 to 6 months in children, adolescents, and young adults of each tumor size, as well as in adults with large tumors. In small tumors with stable tumor growth and (excellent/good) hearing, these intervals can be extended to 12 months. Remarkably, NF2 patients with an ongoing off-label treatment with the VEGF (vascular endothelial growth factor receptor) inhibitor bevacizumab should strictly follow these intervals to rule out possible side effects such as intracranial bleeding events.

The tumor volume and growth rate of VS can be determined either by one-dimensional (1D) linear measurements of the greatest diameter or by three-dimensional (3D) volumetric analysis^6,7^. The disadvantage of the latter procedure is primarily the complexity of its implementation, which takes considerably more time. The development of semi-automation has significantly improved this. Most studies evaluating the growth behavior of NF2-associated VS are based on 1D-based methods, as this is quicker and easier to carry out. However, these seem too imprecise compared to 3D volumetry and have a high intra-observer variability^8^. Furthermore, they are known to underestimate small and overestimate large tumor volumes^6,8^. In addition, previous studies are difficult to compare to each other and our study due to the low numbers of investigated MRI scans in both sporadic^7–9^ and NF2-associated^6,9,10^ VS (*n* = 43–252).

In the clinical routine, classification systems such as the Koos^11^ and Hannover classification^12^ are still used and helpful to give an impression of the tumor size. These stratify the tumors based on intra- and extrameatal extension and brainstem compression. However, these do not give a detailed impression of the growth behavior. Above all, they are often unable to depict and assess small increases or decreases in volume and, thus, possible therapeutic influences.

Unlike sporadic unilateral VS, the bilateral presence of these tumors poses a challenge for all radiological evaluations and volume determinations. Due to the often simultaneously occurring collision tumors such as meningiomas or non-vestibular schwannomas, these are not always easy to differentiate. In addition, they frequently change their configuration and volume due to (often multiple) treatment(s), especially after previous partial resections.

In the following study, we investigated the accuracy and compared 1D and 3D-based methods for determining tumor volume in NF2-associated VS in a large patient/tumor collective. We also referenced and correlated the Koos and Hannover classification systems. In addition, the influence of surgery to reduce the tumor volume on the validity of the measurement methods should also be considered.

## Materials and methods

### Patients and methods

The diagnosis of NF2-related schwannomatosis was confirmed in all patients by clinical evaluation using the updated diagnostic criteria^13^. A total of 149 NF2 patients and 292 tumors were included in this retrospective analysis and were followed up between 2004 and 2021 at the Department of Neurosurgery and Centre of Neurofibromatosis and Schwannomatosis in Tübingen. All procedures and methods performed in this study were in accordance with the ethical standards of the institutional and national research committee and with the 1964 Helsinki Declaration and its later amendments or comparable ethical standards. Informed consent was obtained from all participants or their parents/legal guardians included in the study. Ethical approval was obtained from the ethics board of the Medical Faculty and the University Hospital of Tübingen (No 018/2019BO2, final approval date 17/01/2019).

Five patients exhibited unilateral VS (no contralateral VS visible in the thin-layered MRI at any time point), whereas the remaining 144 patients had bilateral lesions. All included patients had at least two MRI scans and one year of routine radiological follow-up. Both internally and externally performed MRI data sets were used. All patient data and imaging were located on a secure server that is accessible to the clinical staff of the University Hospital Tübingen.

MRIs at any treatment timepoint (observation, before and after surgery, with neoadjuvant or adjuvant (postop) off-label treatment with Bevacizumab and after radiation treatment) were included. The analysis explicitly did not address hearing outcomes or growth behavior or examine the outcome of undergone treatment modalities (surgery, off-label treatment with Bevacizumab, radiosurgery). However, volume changes and the challenges related to measurement methods should be explicitly assessed postoperatively, as (partial) resection often leads to significant alterations in the configuration of the residual tumor, unlike radiation or bevacizumab therapy.

The entire tumor, including its extension inside and outside the internal auditory canals, was measured in all measurement procedures. MRI data sets with implants (cochlea or auditory brainstem implant) were excluded. Tumor size was classified by the Hannover^12^ and Koos classification systems^14^.

For the 3D-based segmented volumetric analysis (SVA), postcontrast thin-sliced (≤ 3 mm) T1-weighted magnetic resonance (MR) sequences were uploaded into the iPlan Net software (Brainlab, Feldkirchen, Germany), and volumes were measured manually or in combination with semiautomated segmentation according to our previous studies^15^.

For the 1D-based linear measurements, the MRIs were first carefully analyzed to visualize the most ideal and most considerable tumor extension (VS width) in axial and coronal sequences. Subsequently, the maximum linear (tumor) diameter (MLD) in axial and coronal (medial to lateral and caudal to rostral/cranial, in total four measurements per MRI) planes were measured according to the RECIST guidelines^16^.

To compare tumor volumes using one-dimensional (1D) and three-dimensional (3D) methods, we employed the cubed maximum linear diameter (MLD³), as previously described^6,7^. Additionally, to give a second volumetric impression, the maximum linear diameters in the anteroposterior, craniocaudal, and transverse planes were multiplied (orthogonal analysis, OA) according to previous studies^7^.

**Fig. 1:**
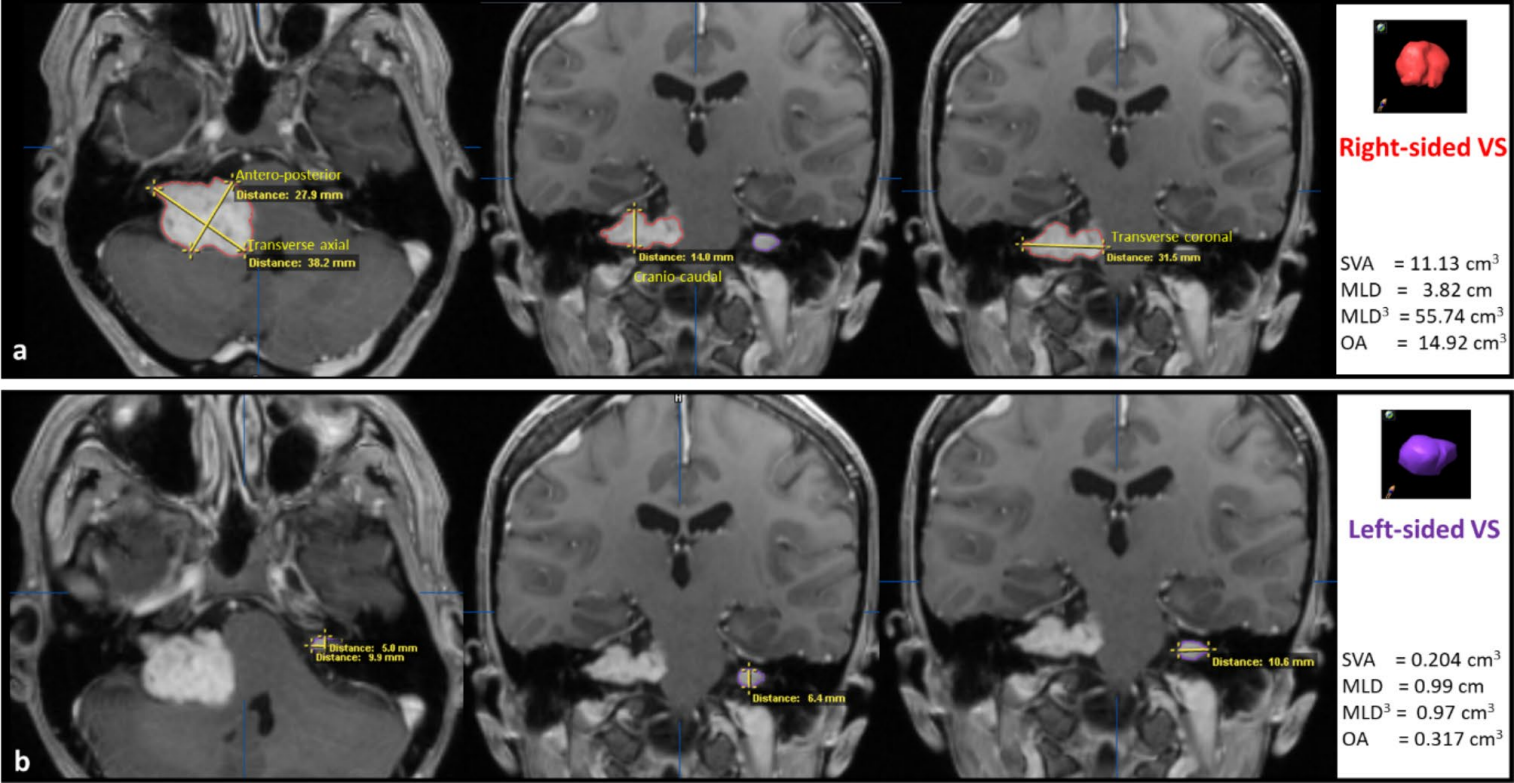
Segmented volumetric analysis (SVA) and linear measurements in (**a**) a right- and (**b**) left-sided NF2-associated vestibular schwannoma. T1-weighted contrast-enhanced, thin-sliced (1 mm) MRI in axial (left) and coronal (middle and right) planes. In the SVA (iPlan Net Software, Brainlab, semiautomated and manual segmentation), the external contours of the right (red color) and left (violet color) are traced, and 3D-volume is assessed (3D-model on the right side in the appropriate tumor color. Tumor diameters were measured in anteroposterior, transverse, and craniocaudal dimensions in axial and coronal planes (defined as “T1 ax width” = anteroposterior and “T1 ax width” = transverse tumor diameter in the axial plane, and “T1 cor width” = craniocaudal tumor diameter in the coronal plane). The greatest linear measurement was defined as the Maximum Linear Diameter (MLD), which was additionally cubed for the volumetric analysis. The maximum linear measurements in the anteroposterior (transverse), cranio-caudal (coronal), and transverse (axial plane) were multiplied to give a second volumetric estimate in the Orthogonal Analysis (OA). The MLD and OA were defined and calculated similarly to previous studies for better comparison. In addition, we have included a fourth dimension (defined as “T1 cor length” = transverse tumor diameter in coronal planes) separately to gain a comprehensive impression.

These represent the tumor’s full extent and are comparable with other studies. The measurement techniques included are illustrated in Fig. 1.

### Statistical analysis

Statistical evaluation was performed using the Statistical Package for Social Studies (SPSS 10.0 for Windows, SPSS Inc.). The significance level was set to a *p-value ≤ 0.05* for all analyses.

To obtain a size reference for the measured and calculated volumes, these were (sub)grouped using the Hannover and Koos classification systems.

A linear regression model was performed to investigate the following aspects/comparisons:

1. Comparison between linear-based diameter assessments (maximum linear diameter, MLD) and their 1D-based volume calculations (orthogonal analysis, OA; cubed maximum linear dimensions, MLD^3^) for segmented (3D) volume (segmented volumetric analysis, SVA).
2. The percentage deviation of the linear-based methods compared to segmented volumetric analysis.
3. The effect and the comparability of OA and MLD^3^ versus SVA methods in terms of postoperative volume reduction through partial resection dimensions.
4. The validity of the classification systems (Hannover and Koos) compared to the linear-based and 3D-based methods.

A graphical analysis of the scattering widths and comparisons of all measurement methods was carried out using plots.

A one-way multivariate analysis of variance (MANOVA) was run to determine the deviation of MLD^3^, OA, and SVA on classification systems Koos and Hannover (estimation grade). The three measurement techniques, MLD^3^, OA, and SVA, were used.

## Results

### Patients/tumors characteristics

Table 1 summarizes detailed demographic and clinical data. In total, 2586 measurements (3D volumetrics: 2586; MLD measurements: 10344, including all dimensions) were performed, and the mean radiological follow-up was 122 (± 83, range 12–448) months.

**Table 1:** Demographic data of 292 neurofibromatosis type 2 (NF2) related vestibular schwannomas (149 patients).

Sex (Number of female/male)	79/70
Operation side (left/right)	146/146
Total radiological follow-up	Mean ± SD, range in months 122 ± 83, 12–448
Age at the time of - First MRI - Last MRI - 1st surgery - 2nd surgery - 3rd surgery - Beginning with off-label BEV	Mean ± SD, range in years 23.1 ± 11.0, 1–59 32.2 ± 13.1, 6–76 23.3 ± 9.3, 8–57 31.2 ± 11.8, 15–73 35.9 ± 10.5, 20–48 25.83 ± 11.21, 15–58
Treatment modalities * - Only “wait and scan” - Surgery (total) - Surgery & BEV - Only off-label BEV - Radiation	Number of tumors (%) 67 (23%) 157 (54%) 54 (18%) 14 (5%) 13 (4%)
Segmented (3D) tumor volume At first MRI (independent of treatment) At last MRI (independent of treatment) *Significance* Before 1st surgery After 1st surgery *Significance* Before 2nd surgery After 2nd surgery *Significance* Before 3rd surgery After 3rd surgery *Significance*	Mean ± SD, range in cm^3^ 3.78 ± 7.73, 0-58.83 2.99 ± 5.98, 0–58.9 *p* = 0.273 7.0 ± 9.82, 0.09–50.86 2.58 ± 6,147, 0–58.83 *p <* 0.001 12.79 ± 13.68, 0.13–65.4 2.87 ± 3.64, 0.03–18.93 *p <* 0.001 6.88 ± 4.36, 0.91–13.13 2.68 ± 2, 1.21–6.08 *p =* 0.090
Cubed maximum linear diameter (MLD^3^) At first MRI (independent of treatment) At last MRI (independent of treatment) *Significance* Before surgery After surgery *Significance*	Mean ± SD, range in cm^3^ 16.45 ± 26.09, 0.05–155.89 15.28 ± 18.24, 0.21–142.48 *p* = 0.279 29.79 ± 34.81, 0.36–193.6 15.67 ± 20.56, 0.05–107.38 *p* < 0.001
Orthogonal analysis (OA) At first MRI (independent of treatment) At last MRI (independent of treatment) *Significance* Before surgery After surgery *Significance*	Mean ± SD, range in cm^3^ 9.65 ± 18.59, 0–123.3 7.72 ± 9.85, 0–54.06 *p* = 0.071 18.28 ± 24.52, 0–123.3 7.36 ± 10.95, 0–68.78 *p* < 0.001
Resection amount categories at 1st surgery 1) Only bony decompression of the IAC 2) Decompression of the IAC with laser coagulation (< 10%) 3) Partial (< 10% to < 90%) 3a) < 10% 3b) ≥ 10% to < 30% 3c) ≥ 30% to < 50% 3d) ≥ 50% to < 70% 3e) ≥ 70% to < 90% 4) Subtotal (≥ 90% to < 95%) 5) Near total (≥ 95% to < 1 00%) 6) Total (100% including tumor capsule) 7) Growth progression No data was available	Number of tumors 0 0 87 11 19 19 10 28 10 19 5 16 20

SD—standard deviation; BEV— Bevacizumab (vascular endothelial growth factor inhibitor); MRI—magnetic resonance imaging; IAC— internal auditory canal. Preoperative and postoperative values were measured within 3 months before and after surgery. * Many patients/tumors underwent several treatment modalities. Of the 157 operated tumors, 114 tumors (73%) were operated on once, 38 tumors (24%) twice, and five tumors (3%) three times. Off-label use with Bevacizumab before or after surgery. Out of the 13 tumors that were irradiated, two were solely treated with radiation. The other 11 tumors underwent a combination of radiation, surgery, and, in some cases, additional Bevacizumab.

The majority of tumors (77%, *n* = 225) had to undergo solid or multiple treatment modalities, whereas 67 tumors (23%) were only observed. Surgery was the primary (70%, *n* = 157/225) treatment of choice, followed by solid or (neo-)/adjuvant off-label treatment with Bevacizumab (30%, *n* = 68), and only 13 tumors were irradiated (6%, *n* = 13). Among 137(157) operated tumors with available MRI scans directly before and after surgery, 87(63%) tumors were partially (10–90% resection extent) resected due to preserving hearing function, and in 34(25%) tumors higher resection extents (> 90%) could be achieved due to preoperative deafness. The remaining 16 operated tumors (12%) even showed a growth progression in the first postoperative MRI after three to six months.

### Strong positive correlations between all included measurement techniques

For the statistical analysis, SVA was defined as the dependent variable (cm^3^) and MLDs (T1ax length, T1ax width, T1cor length, T1cor width), MLD^3^, OA, Koos, Hannover) as independent variables. A total of 2333 measurement values for each technique could be included. All eight variables added statistically significantly to the prediction.

A linear regression model established that all measurement techniques and classification systems (MLDs in coronal/axial planes, MLD^3^, OA, Hannover, and Koos) statistically significantly predict SVA, F (8,2324) = 2003.457, *p* < 0.0005. Residuals were independent, as assessed by a Durbin-Watson statistic of 1.794. Homoscedasticity was evaluated by visual inspection of a plot of standardized residuals versus standardized predicted values. Residuals were normally distributed as assessed by visual inspection of a normal probability plot.

A Pearson’s product-moment correlation was run to assess the relationship between the dependent and independent variables. There was a strong positive correlation between all parameters, *r* > 0.5, and all comparisons were statistically (highly) significant (*p* < 0.001). The correlations of parameters are summarized in Supplementary Table ST1.

**Fig. 2:**
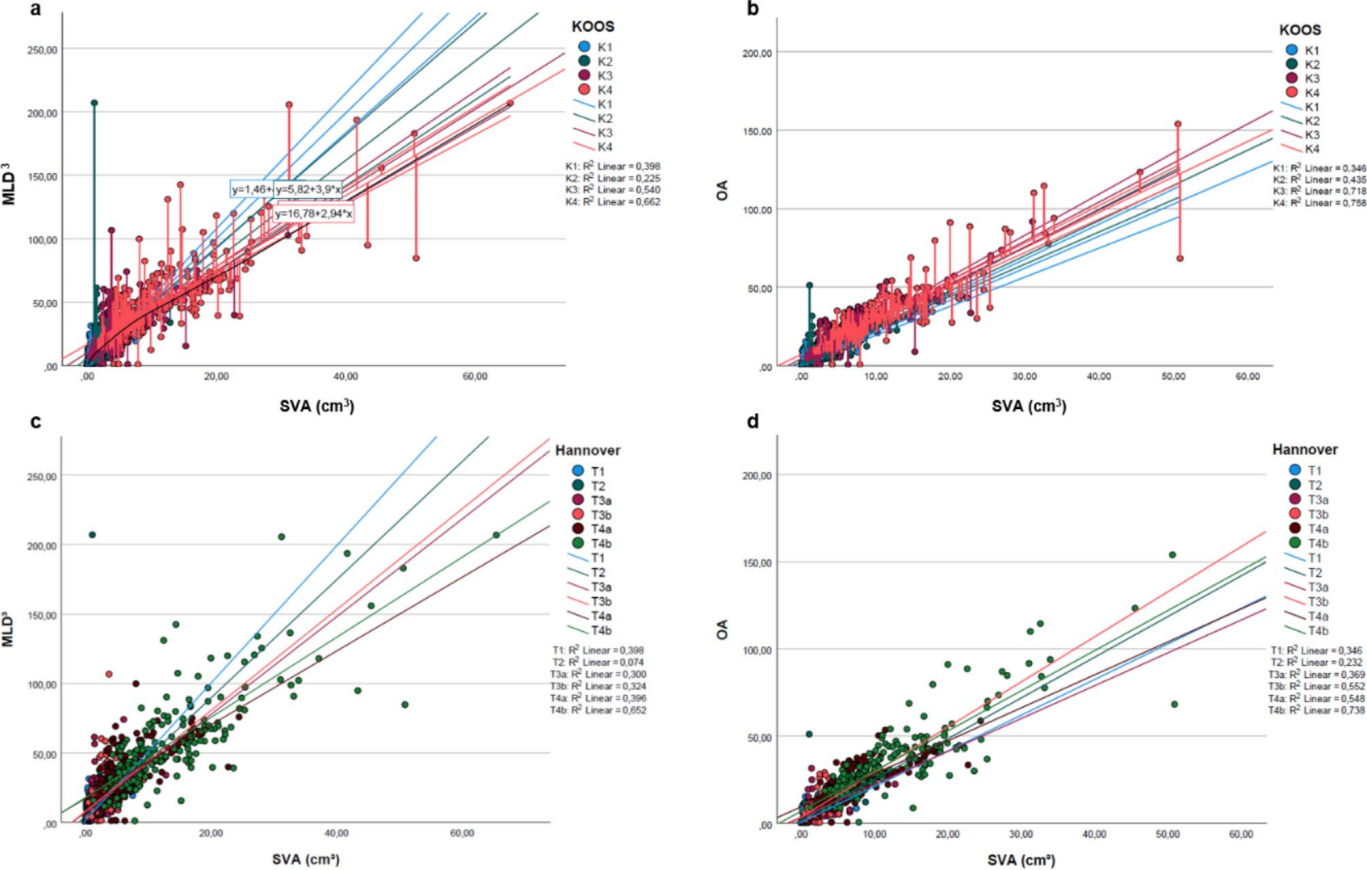
Linear regression model between MLD^3^ / OA and SVA for Koos (**a**,** b**) and Hannover (**c**,** d**) categories. MLD^3^— Cubed maximum linear diameter (MLD^3^) in cm^3^; SVA—segmented volumetric analysis in cm^3^. OA— Orthogonal analysis in cm^3^; SVA—segmented volumetric analysis. Tumor sizes are sub-grouped according to the Koos (K1-K4)^11^ and Hannover classification system (T1, T2, T3a/b, T4a/b)^12^.  The graph describes each Koos/Hannover classification’s correlation coefficient “R2 Linear” values. There is a positive correlation between the two comparative variables for all comparisons. Overall, the correlation between OA and SVA is more robust than between MLD^3^ and SVA, but the scattering range is extensive in both comparison groups.

Since SVA is considered the gold standard and comparative value, the OA showed a stronger positive correlation than the MLD^3^. Within the subgroups according to the classification systems Koos and Hannover, smaller tumors (T1/T2, K1/K2) exhibited a low to moderate positive correlation (SVA vs. OA/MLD^3^: r^2^ = 0.225–0.435) compared to medium-sized (T3, K2, K3) and large tumors (T4, K4) (SVA vs. OA/ MLD^3^: r^2^ = 0.540–0.758). Detailed correlation coefficients are integrated in Fig. 2.

### Large variability/scattering in all tumor sizes with independent variables compared to SVA

Since SVA is defined as the gold standard method, the other measurement techniques exhibited a large variability, especially within small classified tumors (T1, T2, K1). Figure 2 illustrates scattering plots and r^2^-values of the subgroupings in each comparison.

### Influence of a surgically induced tumor volume reduction on 1D (MLD3 and OA) and 3D-based (SVA) volume calculations

**Fig. 3:**
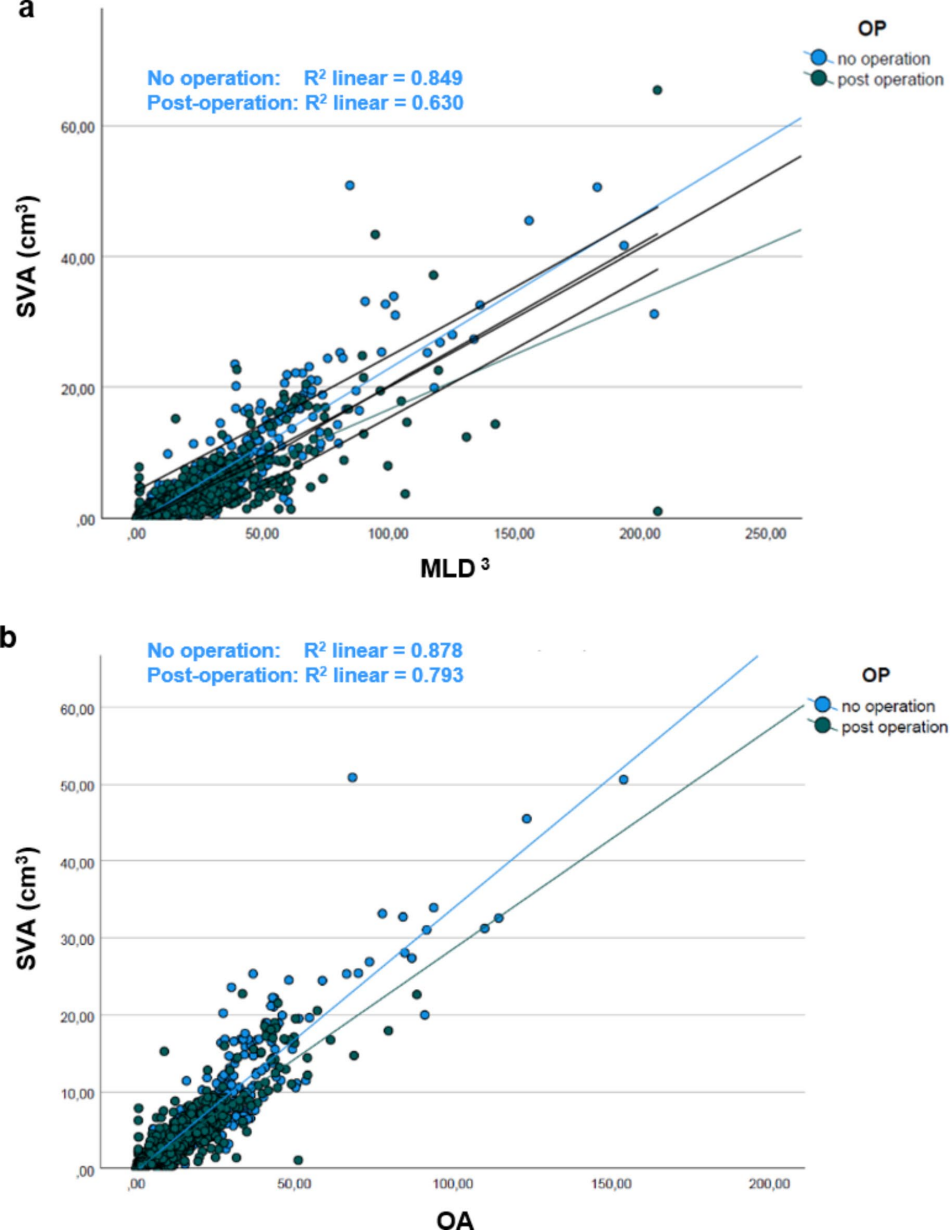
Scattering plots and linear regression for the perioperative comparisons MLD^3^**(a)** and OA **(b)** versus SVA. MLD^3^— Cubed maximum linear diameter (MLD^3^) in cm^3^; SVA—segmented volumetric analysis in cm^3^. OA— Orthogonal analysis in cm^3^; SVA—segmented volumetric analysis.

As the disease usually leads to further growth of the remaining tumor after surgery, subsequent monitoring of tumor growth using measurement methods is essential and decisive for the initiation of further therapeutic measures (usually off-label Bevacizumab or secondary surgery). Due to the usually only partially achievable extent of resection to preserve the hearing, the tumor configurations are generally changed by surgery. A linear regression model established that pre- and postoperative MLD^3^ and OA statistically significantly (*p* < 0.0005) predict SVA (preoperative), F (2,1080) = 3930,627 and SVA (postoperative) F (2,1251) = 2522,296. There was a strong positive correlation between all parameters, *r* > 0.5, and all comparisons were statistically (highly) significant (*p* < 0.001). Residuals were independent, as assessed by a Durbin-Watson statistic. Homoscedasticity was evaluated by visual inspection of a plot of standardized residuals versus standardized predicted values. Residuals were normally distributed as assessed by visual inspection of a normal probability plot. Nevertheless, in the direct comparisons of pre-and postoperative correlation coefficients (scattering plots are illustrated in Fig. 3a and b), postoperative correlation coefficients are weaker than preoperative, particularly in comparing MLD^3^ vs. SVA.

### Percentage deviation of 1D-based volume calculations (MLD3 and OA) and classification systems from 3D volumetry (SVA)

The percentage deviation from MLD^3^ and OA to SVA was normally distributed for each Koos category, as assessed by Shapiro-Wilk’s test (*p* < 0.05). There was a linear relationship between MLD^3^ and OA, as assessed by scatterplot. Koos categories scored higher (overestimation) in MLD^3^ than in OA measurements *(Supplementary Table ST2).* The lower the category/, the smaller the scored tumor size, the higher the (over)estimation in both MLD^3^ and OA, but especially in MLD^3^.

### Group differences (SVA, MLD³, and OA) within the classification systems

The difference between SVA, MLD^3^ and OA on the combined dependent variables *(Koos categories*,* Supplementary Figure SF1a)* was statistically significant, F (6, 4650) = 24.614, *p* < 0.001; Wilks´λ = 0.939; partial η² = 0.031.

Tukey post-hoc tests showed that for SVA and MLD^3^, all comparisons of Koos categories with each other showed statistically significant differences (*p* < 0.05). The most considerable difference in MLD^3^ mean scores was between categories K1 and K4.

For OA, Tukey’s post-hoc test showed that Koos grade K2 showed no significant difference to Koos grade K3 (*p* > 0.05). All other comparisons were statistically significant (*p* < 0.05), and an enormous difference in OA mean scores was seen between categories K1 and K4.

The difference between SVA, MLD^3,^ and OA on the combined dependent variables *(Hannover categories*,* Supplementary Figure SF1b)* was statistically significant, F (15, 2329) = 210.172, *p* < 0.001; Wilks´λ = 0.332; partial η² = 0.307. Tukey post-hoc tests showed that for SVA, MLD^3,^ and OA, all comparisons of Hannover categories with each other showed statistically significant differences (*p* < 0.001).

### Challenging cases for measurement techniques and classification systems

Due to the bilateral presence, the different bilateral tumor sizes, the often presence of adjacent collision tumors, which are not always clearly separable from each other, and surgically induced changes (opening of the internal auditory canal and partial intra- and extrameatal resection amounts), measurement conditions are difficult, especially for linear-based methods and classification systems.

**Fig. 4:**
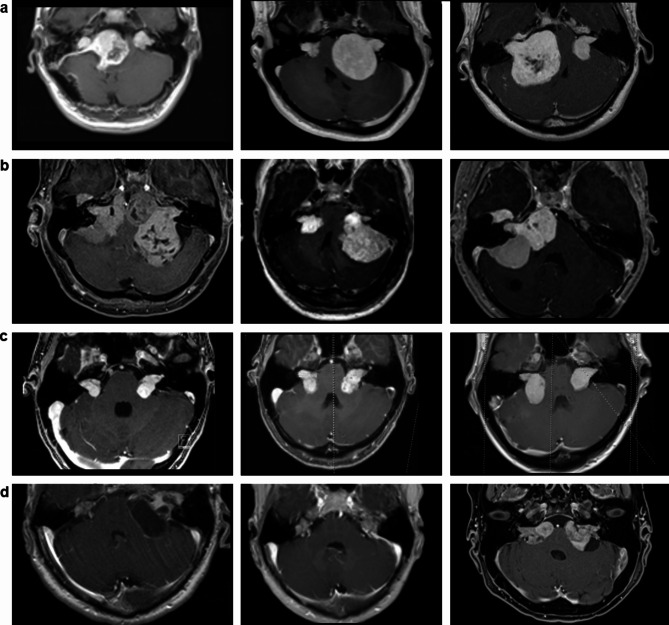
Examples of difficult measurement conditions in NF2-associated VS. T1-weighted contrast-enhanced axial magnet resonance images (MRI) of different NF2 patients. Row a demonstrates different cases and challenges for 1D/3D measurement techniques and classification systems. Rows a and b are separated into three different cases in each column. Row c demonstrates an NF2 case in the follow-up between 2013, 2016, and 2021. Row d illustrates the NF2 case before (first image), after (second image), and postoperatively under Bevacizumab (third image). (**a**) A giant ipsilateral VS often leads to an overestimation in the contralateral side classification systems due to displacement of the anatomical structures (cerebellopontine cisterns, brainstem). For instance, a regularly classified T2 tumor changes into a T3b/T4a tumor due to a large contralateral VS with brainstem compression of the other side, as demonstrated in three different NF2 cases. Large tumors change the configuration and the anatomical conditions between the relevant structures: the internal auditory canal, cerebellopontine cisterns, brainstem, and fourth ventricle. Highly subdivided classification systems, such as the Hannover classification, reach their limits here and are then dependent on the investigator. (**b**) Adjacent collision tumors are frequently found in severely affected NF2 patients throughout the disease and represent a challenge not only for the measurement techniques but also in their differentiation from VS and the subsequent therapy. In the case of collision tumors, meningiomas and adjacent non-vestibular schwannomas cannot always be entirely radiologically differentiated from the VS, and it is not uncommon for a smooth transition to occur. Semiautomatic volumetry has difficulty considering adjacent tumors, so such cases often must be performed entirely by hand. (**c**) Different growth directions also lead to anatomical shifts and make it challenging to classify sizes using classifications as demonstrated in an NF2 case under observation during the period (from left to right) 2013, 2016 to 2021 with a dorsal growth direction on the left side. (**d**) Cystic/regressive tumor components can occur spontaneously (first two images) and in response to Bevacizumab (last image). The first demonstrated case was operated on an expansive and rapidly growing tumor cyst on the left side. Under hearing preserving criteria, the solid tumor parts could be just marginally resected. Therefore, the solid tumor size did not change in its volume. The question is whether cysts should be considered in tumor volumetry, especially in monitoring bevacizumab therapy. They usually represent an excellent response to Bevacizumab and should not be regarded as volume addition in 3D volumetry. This naturally makes SVA more complex, and linear-based measurements cannot consider such changes if they occur centrally in the tumor and not in peripheral areas.

**Fig. 5:**
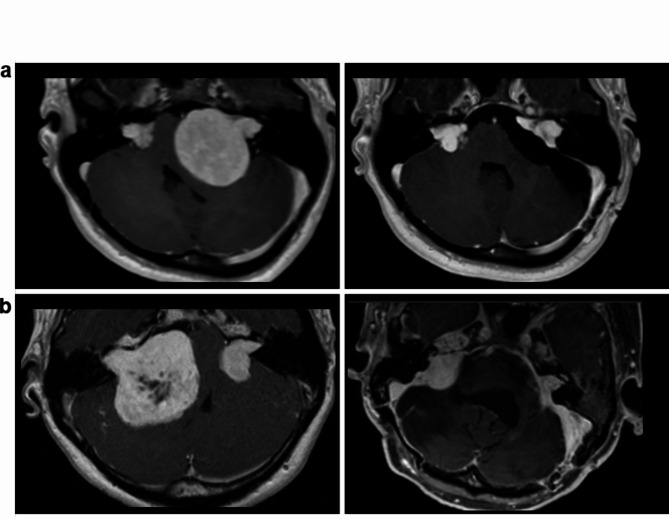
Exemplary cases of surgically induced anatomical changes and limits of the classification systems. T1-weighted contrast-enhanced axial magnet resonance images (MRI) of different NF2 patients. Two (cases a and b) other and operated NF2-associated vestibular schwannoma (VS) cases. The left column shows the preoperative, and the right column shows the postoperative images. (**a**) Bilateral VS, preoperative tumor size left-sided T4b/K4 and non-operated right-sided T3b/K3 VS. Surgery of the giant, left-sided VS (T4b/K4) VS was indicated and performed (regardless of hearing) due to displacement of the brainstem and fourth ventricle. A slight tumor residue had to be left to preserve the facial nerve function. The internal auditory canal (IAC) was also opened. According to Hannover, postoperative classification into T1/T2/T3a or Koos into K1/K2 is more complicated. The contralateral right side became a preoperative T4a to a “postoperative” T3a due to the relief of the brain stem. The Koos grade K2 did not change due to surgery. (**b**) Bilaterally operated VS, preoperative tumor size right-sided T4b/K4 and left-sided T4a/K3. The relief of the brain stem resulted in an altered configuration of the posterior fossa with almost compensatory widening of the fourth ventricle. Due to this shift in the anatomy after partial resection of the large and the opening of the internal auditory canal, it is tough to classify tumors in classification systems on both sides. This case also shows the problem of the different tilting of the MRI diagnostics.

This often leads to shifts in the structures in the area of the internal auditory canal and the cerebellopontine angle. In addition, the growth directions could be different, which is challenging, particularly for the classification systems. Figures 4 and 5 illustrate these difficulties.

## Discussion

In recent years, 3D segmented volumetric analysis has become increasingly important in the monitoring and treatment surveillance of NF2-associated vestibular schwannoma. This method is indispensable, especially in the monitoring and control of off-label therapy with Bevacizumab. Above all, this method is very time-consuming. Due to this, many centers still assess tumor volume using 1D-based measurements and volume calculations. Previous studies investigating this topic are very heterogeneous and complex to compare with each other and our study.

In these studies, the number of analyzed MRI scans is lower (43[6], 252 [8], 101 [17], 115/230 [18]), the linear measurement methods are different^6^, and most of them investigated sporadic VS^17^ rather than NF2-associated tumors^6,9,10,18^ were performed. Mainly, these studies showed a high intra-observer variation^8^, an underestimation of the volume of small tumors^8,9^, and an overestimation of the volume of large tumors^8,9^ by linear-based volume calculations. Also, only a few follow-up data of a maximum of 12 months were taken into account^17^ (Vokurka et al.: 38 patients with two MRIs within 12 months FU), and intervals between MRIs were larger (average 2.6 years^7^).

Our study features a larger cohort of NF2 patients (149) and VSs (292), more measurements, and a more extended follow-up period (averaging 122 months, approximately ten years) than previous studies. This broader scope enhances the clarity and validity of our findings regarding measurement techniques for monitoring NF2-associated vestibular schwannomas. We have decided to determine the 1D-based measurements MLD, MLD^3^, and OA analogous to the study by Walz et al.^7^, as this also allows sufficient measurement of the tumor extent in the internal auditory canal (IAC). This was usually not included in the other studies, and also small tumor volumes were excluded (< 1cm^3^)^10^. Including the tumor component in the IAC is essential for assessing NF2-associated tumors, as small tumors are often initially present, particularly in children, and require close monitoring. This is most likely to explain the overestimation of small tumors and underestimation of large tumors in linear-based measurements in our data and the data of Walz et al.^7^. In addition, we have a large amount of data with a close (the average interval between MRIs was 3–6 months), and the long-term radiological follow-up gives a more detailed impression of the growth behavior and volume change. Slice thickness in the studies was similar and less than 3mm^7^. It has to be considered that the accuracy of linear measurements is exposed to more interference factors, such as the effect of slice thickness and different patient repositioning from scan to scan^19^.

Our linear-based measurements assume a cubic rather than an ellipsoid tumor configuration, which may further explain the different estimates between the studies. Harris et al.^6^ are also based on cubic measurements and, in contrast to us, have detected an underestimation of small tumor volumes.

Segmented volumetric analysis (SVA) limitations include the time required for measurements, especially in NF2 patients. This is due to the bilateral presence of VS, the frequent occurrence of other adjacent (collision) tumors like non-vestibular schwannomas and meningiomas, and the often large tumor volumes. These complexities can complicate accurate and timely evaluation. Despite the semiautomatic procedures, manual segmentation is often required to differentiate VS from adjacent collision tumors, particularly the tumor mass in the IAC. Accurate tumor volume determination requires a slice thickness of < 3 mm and at least five slices^20^. A high investigator experience is essential for precise measurements, which can be problematic when using external MRI with varying protocols in smaller centers that lack adequate resources(e.g., software). Determining the volume of vestibular schwannomas, especially in cases with adjacent collision tumors, can take 20 to 30 min on average, leading to a total analysis time of nearly one hour for complex NF2 cases with large tumors. Integrating this into a clinical routine without additional staffing is not feasible, and health insurance does not cover the costs. Nevertheless, it reflects the volume in the most significant detail. It considers all growth directions and small volume changes, particularly important in monitoring off-label treatment with Bevacizumab.

The colleagues of Morris et al.^10^ detected a sensitivity of 86% for linear measurements compared to volumetric MRI measurements in 61 NF2-associated VS on Bevacizumab using a 2-mm threshold to define a tumor progression. They pragmatically recommended that tumor volumetry be completed in cases with a 2-mm change in linear measurements. In advance, they excluded tiny volumes (< 1cm^3^) from the analysis. Since Bevacizumab medication has a poor response in small/surgically reduced VS and in children and adolescents (who usually have small tumors)^21^, it is mainly used for medium-sized/large tumors. This is a pragmatic approach, also taking into account our results, as we were also able to demonstrate a very high variability in small tumors.

Innovative functional MRI techniques have been described for assessing the effectiveness of treatments, providing a valuable complement to tumor growth evaluations. In the research conducted by Li et al., biomarkers obtained from dynamic contrast-enhanced MRI (DCE-MRI) were found to predict the volumetric response of vestibular schwannomas (VS) to Bevacizumab therapy^5^. A separate study by Özer et al. demonstrated that DCE-MRI may be effective in monitoring and predicting treatment responses in VSs after undergoing radiosurgery^3^.

Classification systems provide a general estimate of tumor size but lack precision in evaluating therapy and monitoring growth in NF2-associated vestibular schwannomas (VS). They primarily focus on growth towards the brainstem and overlook directions. Depending on the examiner, they are still subject to fluctuation ranges and are difficult to delineate, particularly in their transitions, especially in the Hannover classification (T2 in T3, T3b in T4a). However, they can be quickly obtained and provide a sufficient rough estimate of tumor size for everyday clinical practice. The relationship between classification systems and 3D segmented volumetric analysis or linear-based methods has not been adequately investigated. Except for relevant, often bilateral brainstem compression (T4, K4), the hearing status is always the main factor in treatment decisions or monitoring for NF2 patients. Consequently, classification systems are secondary in managing small and medium-sized tumors (T1-T3 and K1-K3).

Of course, our study also has its limitations. Firstly, the analysis’s retrospective nature and single-center design may introduce biases. The lack of uniformity in MRI performance could lead to inconsistent data collection. Additionally, a standardized protocol, which is typically expected in a prospective analysis, was not implemented in this study. This variability could affect the comparability and generalizability of the findings regarding measurement techniques used for NF2-associated VSs.

Furthermore, many MRI and hearing diagnostic follow-ups occur close to patients’ homes without a standardized protocol, with the results sent to our center for further assessment. This leads to variable slice thickness (although we have defined a cutoff of < 3 mm slice thickness) and differences in patients’ positioning. Nevertheless, to minimize the external variability of the implementation, patients were encouraged to have the MRI performed at the same institution close to home.

Second, the criteria for including MRI diagnostics were clearly defined as a thinly layered T1 sequence with contrast medium in axial and coronal planes. MRI data sets with CI or ABI were excluded. One examiner performed the linear measurements, and a maximum of two very experienced examiners performed the 3D volumetry.

The data under therapy were included deliberately as most NF2 patients will require (multiple) treatment(s) throughout their lifetime. However, the treatment effect of the individual groups (observation, surgery, Bevacizumab treatment, and radiation) was not evaluated in the present study, as the focus was on comparing the measurement methodology. The volume changes caused by an operation were explicitly selected because they changed the tumor configuration the most. The differences between the measurement procedures after radiotherapy or under Bevacizumab are not significantly different.

Automated volumetric measurements with deep learning networks would significantly facilitate the clinical workflow in NF2 patient care. This has only been tested for sporadic vestibular schwannoma, with heterogeneous results compared to manually segmented/contoured volume determinations^22,23^. The bilateral presence and the often adjacent collision tumors or non-vestibular schwannomas make the application considerably more difficult. Nevertheless, our working group is in the process of testing and training such procedures.

## Conclusion

Volume calculations based on an orthogonal analysis of the maximum diameter represent an excellent alternative to time-consuming segmented tumor volumetry. Nevertheless, these methods are subject to an enormous scatter range and percentage deviation from segmented (3D) volumetry, especially in the case of small tumor volumes that have been reduced natively or surgically. Classification systems are sufficient for categorizing tumors and assessing intra- and extrameatal extension but are not suitable for detailed monitoring of tumor volume. Avoiding deviations in volume determination is essential, especially for monitoring off-label treatment with bevacizumab or treatment decisions (e.g., timepoint of surgery) and postoperative surveillance predominantly in small (T1/2, K1/2) tumors, with adjacent collision tumors, or linear changes > 2 mm. Segmented volumetrics should remain the gold standard here despite the time required.

## Electronic supplementary material

Below is the link to the electronic supplementary material.

Supplementary Material 1

## Data Availability

All available data is included in the manuscript and its Supplementary Information files.
