# Supplementary material for: Comparison of 1D and 3D volume measurement techniques in NF2-associated vestibular schwannoma monitoring
Source: Sci Rep. 2025 Jan 17;15:2313. doi: 10.1038/s41598-025-85386-4 (PMC11742393; doi:10.1038/s41598-025-85386-4)
Supplement: Supplementary file 1 — Supplementary Material 1 [file 41598_2025_85386_MOESM1_ESM.docx]

**Supplementary Materials**

Comparison of 1D and 3D Volume Measurement Techniques in NF2-associated Vestibular Schwannoma Monitoring

Scientific Reports

Isabel Gugel*, Nuran Aboutaha, Bianca Pfluegler, Ulrike Ernemann, Martin Ulrich Schuhmann, Marcos Tatagiba,

Florian Grimm.

*Corresponding author: Isabel Gugel, MD PhD, Department of Neurosurgery, Centre of Neurofibromatosis and Schwannomatosis, Centre of Rare Disease, University Hospital Tübingen, Tübingen, Germany; https://orcid.org/0000-0003-2923-5949; isabel.gugel@med.uni-tuebingen.de.

**Supplementary Table ST1.** Strong positive correlations of all measurement techniques in determining the volume and size of NF2-associated vestibular schwannoma.

| **R-values** | **SVA** | **MLD^3^** | **OA** | **KOOS** | **Hannover** |
| --- | --- | --- | --- | --- | --- |
| **SVA** | 1 | 0.856 | 0.921 | 0.647 | 0.683 |
| **MLD^3^** |  | 1 | 0.927 | 0.696 | 0.722 |
| **OA** |  |  | 1 | 0.699 | 0.732 |

**Note.** R – Pearson´s Correlation Coefficient; SVA – segmented volume analysis; OA – orthogonal analysis; MLD – maximum linear diameter; MLD^3^ – cubed maximum linear diameter; T1 – T1 weighted magnetic resonance images (MRI) with contrast agent; ax – axial planes; cor – coronal planes. Tumor size according to Koos [1] and Hannover [2] classification. All comparisons were statistically significant p < 0.001. A total number of 2333 measurements each could be included in the analysis. Highlighted grey fields represent duplicate values.

**Supplementary Table ST2.** Percentage deviation change from MLD^3^ and OA to SVA for each Koos or Hannover category.

| **Koos Grading** | **Mean MLD^3^ ± SD, range in %, Number (n)** | **Mean OA ± SD, range in %** |
| --- | --- | --- |
| K1 | 1364.87 ± 1818.21, 111.9-18231.13, n = 817 | 621.27 ± 914.67, 53.23-9947.41 |
| K2 | 1003.61 ± 893.73, 127.06-19291.71, n = 711 | 494.92 ± 300.39, 59.68-4770.74 |
| K3 | 795.44 ± 489.51, 17.32-3500.0, n =486 | 445.18 ± 188.93, 10.82-1604.64 |
| K4 | 549.57 ± 243.30, 13.75-1448.66, n =316 | 333.72 ± 115.30, 9.75-909.53 |
| **Hannover Grading** | **Mean MLD^3^ ± SD, range in %, Number (n)** | **Mean OA ± SD, range in %** |
| T1 | 1364.87 ± 1818.21, 111.9-18231.13, n =817 | 621.27 ± 914.67, 53.23-9947.41 |
| T2 | 1143.25 ± 1246.95, 127.06-19291.71, n =280 | 542.76 ± 375.33, 59.68-4770.74 |
| T3a | 1000.82 ± 579.92, 137.11-4404.12, n = 401 | 497.45 ± 252.09, 97.68-2271.43 |
| T3b | 784.26 ± 453.69, 53.53-3500.0, n = 398 | 439.45 ± 175.13, 26.98-1604.64 |
| T4a | 602.55 ± 264.88, 17.32-1566.26, n = 249 | 360.54 ± 120.56, 10.82-909.53 |
| T4b | 487.62 ± 192.34, 13.75-1258.02, n = 185 | 311.22 ± 100.83, 9.75-730.12 |

**Note.** No—Number; SD—standard deviation; MLD^3^— Cubed maximum linear diameter (MLD^3^) in cm^3^; SVA—segmented volumetric analysis in cm^3^. OA— Orthogonal analysis in cm^3^; SVA—segmented volumetric analysis. The numbers of MLD^3^, OA, and SVA are the same. Tumor sizes are sub-grouped according to the Koos (K1-K4) [1] and Hannover classification [2]. Subgrouping K1 and T include the same tumors (intrameatal) therefore the mean values were identical.

**Supplementary Figure SF1.** Boxplot Model of the Multivariate Analysis of Variance in **A** Koos [1] and **B** Hannover classification [2] in SVA (blue color), MLD^3^ (yellow color), and OA (red color).


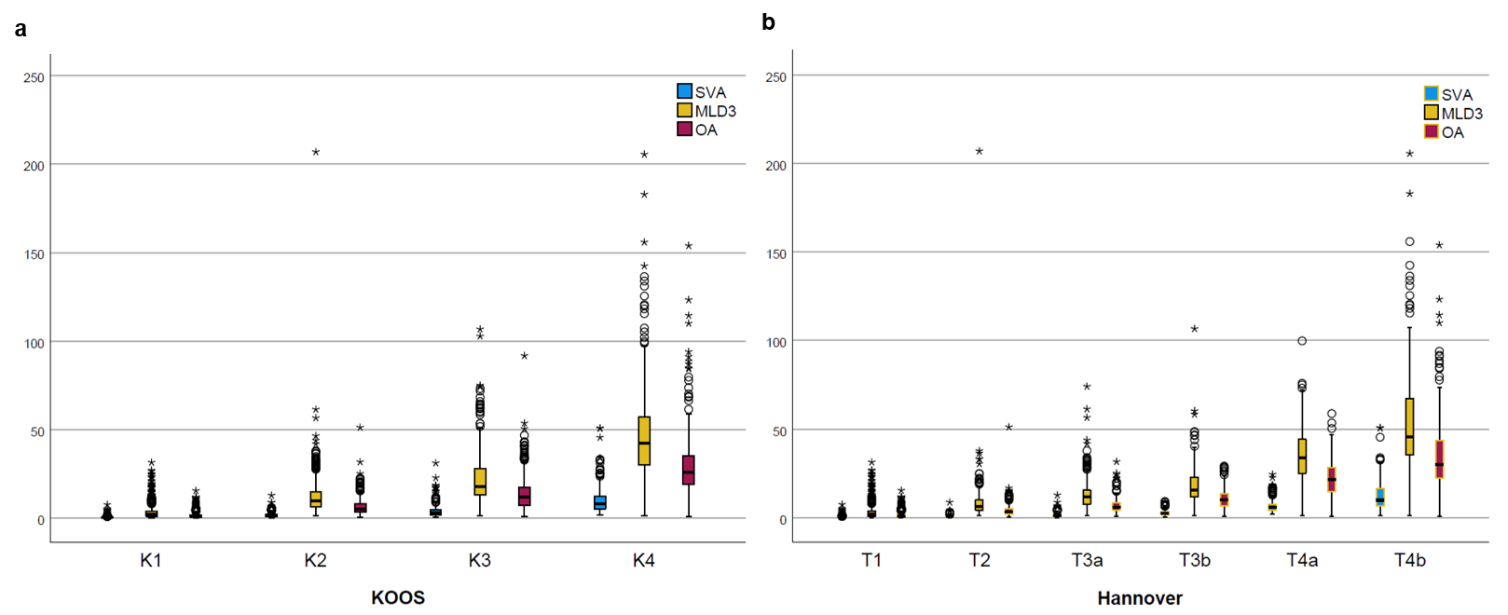


References:

1. Erickson NJ, Schmalz PGR, Agee BS, Fort M, Walters BC, McGrew BM, Fisher WS (2019) Koos Classification of Vestibular Schwannomas: A Reliability Study. Neurosurgery 85: 409-414 doi:10.1093/neuros/nyy409

2. Samii M, Matthies C (1997) Management of 1000 vestibular schwannomas (acoustic neuromas): hearing function in 1000 tumor resections. Neurosurgery 40: 248-260; discussion 260-242
